# Design and Performance Evaluation of TPMS-Based Dual-Layer Gradient Porous Structures for Bone Scaffolds

**DOI:** 10.3390/jfb17030144

**Published:** 2026-03-13

**Authors:** Xiaobing Li, Donglai Zhou, Cuiyuan Lu, Min Zhong, Xianda Xie, Linyu Zhou, Yanghan Fu

**Affiliations:** 1 School of Advanced Manufacturing, Nanchang University, Nanchang 330031, Chinadonglaizhou@gdc.edu.cn (D.Z.);; 2 Modern Mechanical Design Engineering Technology Research Center of Jiangxi Province Higher Education Institutions, Nanchang, 330031, China; 3 School of Mechanical and Electronic Engineering, Gandong University, Fuzhou 344000, China; 4 Jiangxi Province Key Laboratory of Light Alloy, Nanchang University, Nanchang 330031, China

**Keywords:** triply periodic minimal surface (TPMS), dual-layer gradient structure, yield strength, elastic modulus, Permeability, bone scaffold

## Abstract

This study investigates and compares properties of various P-type Triply Periodic Minimal Surface (TPMS) porous structures for bone scaffold design. At first, six cases of homogeneous single/dual-layer structures, axial single/dual-layer gradient structures and radial single/dual-layer gradient structures with the same average porosity are developed. Dual-layer gradient structures are selected for further design due to more similar pore and stress distributions to human bones, reduced maximum stress, higher yield strength and greater variations in yield strength and elastic modulus (*E*). The mechanical and permeability properties of ten cases of axial and radial dual-layer gradient structures with the same overall porosity but different inner and outer layer porosities are then further designed and studied. The results show that yield strength is within 112.75–139.97 MPa, *E* ranges from 11.15 to 13.01 GPa, the permeability (*K*) falls within 1.51–10.01 × 10^−9^ m^2^ and the average wall shear stress (*WSS*_avg_) varies between 6.18 and 9.11 mPa. The yield strength, *E* and *K* of radial dual-layer gradient structures are higher and *WSS*_avg_ is lower than those of axial dual-layer gradient structures. Moreover, with increase in inner average porosity ($\bar{P}$) and decrease in outer $\bar{P}$, the yield strength, *E* and *K* gradually decrease while *WSS*_avg_ gradually increases for both types of structures. In particular, the radial dual-layer structure with the lowest porosity of 27.5% in the inner layer and highest porosity of 42.5% in the outer layer has superior mechanical and permeability properties. The findings offer direct guidance for the structural design of bone implants, enabling performance customization for different applications.

## 1. Introduction

The phenomenon of aging population stimulates the rise in the number of patients with bone defects [1], while luckily bone tissue engineering provides an effective solution by replacing the defective bone with bone scaffolds [2]. Triply Periodic Minimal Surface (TPMS) structures have advantages in bone scaffold design since they have implicit and smooth surfaces with pore interconnections, which are similar to the structure of human bone. Such structures also show excellent mechanical strength and cell responses [3]. As a result, they have the potential to be widely used in the field of bone tissue engineering.

**Figure 1:**
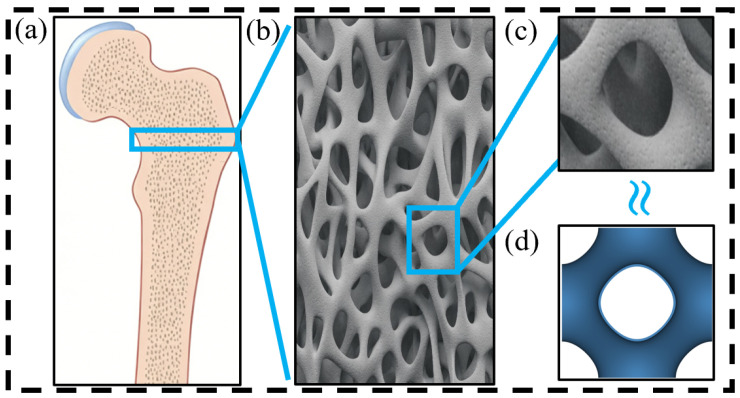
(**a**) Schematic diagram of femur, (**b**) AI-generated illustrative image of femoral tissue, (**c**) higher magnified image of (**b**), (**d**) computer generated P-type TPMS cell structure.

In the field of bone defect repair, porous scaffolds are required to balance mechanical stability at the initial implantation stage with a permeable microenvironment supported by pore interconnectivity that is favorable for bone growth [4]. Although increasing porosity can enhance pore interconnectivity and permeability, thereby promoting nutrient transport and cell migration, it generally reduces the structural stiffness and strength, which compromises the mechanical stability during the early implantation stage [5]. Therefore, homogeneous scaffolds often struggle to satisfy these competing requirements simultaneously. To address this trade-off, porosity-gradient scaffolds have been extensively proposed and validated in recent years. By spatially distributing porosity, various functions can be realized: a low-porosity peripheral region provides cortical bone-like load-bearing capacity and initial stability, whereas a high-porosity inner region offers trabecular bone-like interconnected pathways. As such, an overall structure that more closely resembles the natural cortical–trabecular bone can be realized [6,7]. In addition, the porosity of gradient scaffolds is commonly designed within a 50–90% range to balance early-stage mechanical stability and favorable permeability for nutrient transport, cell adhesion and proliferation, and vascularization, which support bone growth [8]. When combined with pore sizes of approximately 600 to 900 μm, enhanced overall performances can often be achieved [9,10]. For illustration, the schematic diagram of a femur is presented in Figure 1a. The AI-generated image of femoral tissue is shown in Figure 1b, which shows the interconnected porous network. The higher magnified AI-generated illustrative image of the porous structure is displayed in Figure 1c, which presents similar P-type TPMS cell as generated by a computer (Figure 1d).

Most bone scaffolds are made of stainless steel and titanium alloy [11,12], which have higher elastic modulus than human bones, and thus the issue of stress shielding is likely to occur [13], resulting in improper stimulation and gradual loss of human bone mass in the surrounding bone [14,15]. In response, TPMS bone scaffolds should be designed to match the elastic modulus of human cortical bones, which is usually set within 10–30 GPa [16]. The design of bone scaffold also needs to take permeability into account in order to meet the needs of cellular activities as well as material exchange during bone tissue repair and regeneration [17,18,19], and the permeability is typically considered to match that of trabecular bone [20]. Previous studies have shown that the permeability distributions of human bone samples taken from the vertebral body of the calcaneus [21,22], the femoral bone [23,24], and the spine [25,26] range from 2.56 × 10^−11^ m^2^ to 74.3 × 10^−9^ m^2^ [27].

TPMS structures have shown advantages in designing flexibility since they are defined by implicit constitutive equations and thus are relatively easy to manipulate. Studies have been carried out in generating uniform or graded TPMS structures with different porosities and investigating the corresponding properties. Bobbert et al. study the mechanical property and permeability of sixteen TPMS porous structures with four (primitive (P), I-WP, gyroid (G) and diamond (D)) unit cell types and four porosities ranging from 43% to 77%, and obtain a superior combination of relatively low elastic modulus and high yield stress [28]. Liu et al. compare six D-type and G-type uniform and gradient structures with different pore sizes of porosities 60%, 70% and 80%, and they find that uniform D-type structures with large pore size exhibit excellent properties since they own sufficient mechanical properties and high permeability [29]. Verma et al. study the performance of P-type structures and discover that the elastic modulus and compressive yield strength are decreased by about 80% and 76%, respectively, when the porosity is increased from 40% to 80% [30]. Han et al. generate a series of G-type structure with different porosities of 30–70%, and the results show that the elastic modulus and compressive yield strength decrease gradually with increase of porosity [31].

Although diverse TPMS structures have been developed and their properties have been compared in previous studies, the designs and multiple properties of TPMS structures with same average porosity for bone scaffold application are rarely studied or reported. In this study, P-type, which has a similar shape of porous structure to bones, is selected for analysis, and two types of gradient structures: namely, axial and radial structures are both considered. Additionally, novel dual-layer TPMS structures composing inner and outer layers are developed. Specifically, single-layer and dual-layer homogenous, axial gradient and radial gradient structures with the same average porosity are designed at first, and dual-layer gradient structures which have similar gradient porous distribution to human bones and possess less maximum stress and difference in stress distribution are chosen for further design and analysis. Subsequently, ten axial and radial dual-layer gradient structures of the same average porosity but different inner and outer layer porosities are developed. Mechanical properties and permeability properties of those structures are fully studied and evaluated for bone scaffold application, the relationship between the dual-layer gradient structure design and the resulting properties are revealed, and the best dual-layer gradient structure is discovered.

## 2. Methods

### 2.1. Cell Structure

TPMS has a smooth surface with zero mean curvature at all points, and its surface varies periodically in space. Although TPMS can be expressed exactly by the Weierstrass formula, it is commonly represented using the implicit periodic surface approximation. For the P-type TPMS (referred as P-type), the approximated equation $\phi_{P}$ is presented as Equation (1):

(1) $$\phi_{P}(x,y,z)=\cos(\omega x)+\cos(\omega y)+\cos(\omega z)=C$$

where *ω* = 2*π*/*l*, *l* is the period of the surface, *x*, *y*, *z* are the three dimensions of spatial coordinates, and *C* is the bias which controls the shape and area of the surface. The structure surface of $\phi_{P}=C$ divides the space into two regions of $\phi_{P}<C$ and $\phi_{P}>C$. In order to obtain different structures, *C* values need to be set accordingly. When *ω* = 1 and *C* = 0, the obtained P-type cell structure is shown in Supplementary Figure S1.

The porosity (*P*) of the structure can be calculated by the volumetric method, as displayed in Equation (2):

(2) $$P=\frac{V_{0}}{V}\times 100\%$$

where *V*_0_ is the porous volume of the cell structure and *V* is the volume occupied by the smallest enclosing box of the porous cell, as shown in Supplementary Figure S1. When $\phi_{P}<C$ is selected as the solid region, $\phi_{P}>C$ is then the porous region, and vice versa. The porosities of P-type structures need to be set within a certain range. On one hand, if the porosity is too high, pinch-off (Supplementary Figure S2a) will occur, while on the other hand, if the porosity is too low, pore channels will be closed (Supplementary Figure S2b). After testing, the upper porosity threshold for avoiding pinch-off is 78%, while the lower porosity boundary for avoiding closed pore channel is 22%. Therefore, the theoretical porosity range of P-type structure is 22–78%.

In order to find the relationship between porosity and bias *C*, *C* values with intervals of positive and negative 0.2 from *C* = 0 and the corresponding porosities are generated. The bias *C* values, the calculated porosity *p* values, and the fitting lines are presented in Supplementary Figure S3. It can be seen that *P* and *C* are linearly correlated. Such linear relationships can be fitted and formulated as Equations (3) and (4), which are obtained by fitting *P* and *C* when the region $\phi_{P}<C$ and $\phi_{P}>C$ is assigned solid, respectively.

(3) $$P=(-0.2883C+0.5001)\times 100\%\;(\phi_{P}<C)$$

(4) $$P=(0.2882C+0.5003)\times 100\%\;(\phi_{P}>C)$$

### 2.2. Gradient Structure

#### 2.2.1. Axial Single-Layer Gradient Structure Design

When designing gradient structure, axial single-layer gradient structure is proposed first. The structure is developed by changing the bias *C* with respect to the distance along the axial direction (e.g., *x* coordinate) as in Equation (5).

(5) C=ax+b

For axial single-layer gradient structure design, $\phi_{P}<C$ is assigned solid, and an axial length of 10 mm is used. The highest porosity of 78% and the lowest porosity of 22% are set at *x* = 0 and *x* = 10 mm, respectively. The coefficients of *a* and *b* in Equation (5) can be solved based on Equation (3) and the above threshold values. The resulting bias function *C* can be expressed as a function of *x* as shown in Equation (6). The equation of the solid region of the axial single-layer gradient structure is expressed as Equation (7), and the corresponding structure with respect to *x* within 0–10 mm is displayed in Supplementary Figure S4.

(6) C=0.194x−0.97

(7) $$\phi_{P}\leq 0.194x-0.97$$

#### 2.2.2. Axial Dual-Layer Gradient Structure Design

Since a porosity range of 50–90% is generally considered when designing bone scaffolds [8], while axial single-layer gradient structure has theoretical non-defect porosity range of 22–78%, in response, a modified axial gradient structure is generated. The structure is characterized by two axial single-layer gradient structures, and is referred to as an axial dual-layer gradient structure. The structure is developed as follows: one structure, referred to as the outer axial gradient structure, is developed by assigning $\phi_{P}\leq C_{1}(x)$ as solid and $\phi_{P}>C_{1}(x)$ as the porous region, as shown in Supplementary Figure S5a. In contrast, an inner axial gradient structure is formed by assigning $\phi_{P}\geq C_{2}(x)$ ($C_{1}(x)>C_{2}(x)$) as solid and $\phi_{P}<C_{2}(x)$ as the porous region, as displayed in Supplementary Figure S5b. The axial dual-layer gradient structure is then created by a Boolean intersection operation on the above outer and inner axial gradient structures, and the resulting structure is shown in Supplementary Figure S5c. The porosity of the dual-layer gradient structure is the sum of the porosities of the outer and inner gradient structures.

#### 2.2.3. Radial Single-Layer Gradient Structure Design

Radial gradient structure is generated when bias *C* is defined as a continuous function of radial change as shown in Equation (8).

(8) $$C=k\cdot r^{n}+b$$

where *r* is the radius, and *k*, *b*, *n* are three parameters which control the gradient structure. It can be seen that the bias *C* changes radially with *r*. When the radius of the radial gradient porous structure is R, the internal (*r* = 0) porosity is noted as *P*_in_ and the external (*r* = R) porosity is defined as *P*_out_, and the bias *C* values corresponding to *P*_in_ and *P*_out_ are *C*_in_ and *C*_out_, respectively. When *n* is specified, parameters *k* and *b* can be obtained as shown in Equations (9) and (10), respectively.

(9) $$k=(C_{out}-C_{in}){\left(\frac{1}{R}\right)}^{n}$$

(10) $$b=C_{in}$$

For example, when $\phi_{p}$ ≤ *C*(*r*) is assigned as the solid region, the bias function *C* (Equation (11)) is obtained based on Equations (3), (9) and (10).

(11) $$C(r)=\frac{\left(P_{out}-P_{in}\right)}{-0.2883}\cdot {\left(\frac{r}{R}\right)}^{n}+\frac{P_{in}-0.5001}{-0.2883}$$

Substituting Equation (11) into Equation (3) yields the porosity distribution function for radial gradient porous structure as shown in Equation (12):

(12) $$P(r)=\left(P_{out}-P_{in}\right)\cdot {\left(\frac{r}{R}\right)}^{n}+P_{in}$$

When the radial gradient porous structure is a cylinder with radius R and height H, the volume of its porous region is denoted as $V_{1}$; then the average porosity can be calculated by Equation (13):

(13) $$\bar{P}=\frac{V_{1}}{\pi R^{2}H}=\frac{\int_{0}^{R}2\pi rP\left(r\right)Hdr}{\pi R^{2}H}=\frac{n\cdot P_{in}+2P_{out}}{n+2}$$

When $P_{in}$, $P_{out}$, $\bar{P}$ are specified, *n* can be calculated based on Equation (13), and the bias *C* function can be obtained based on Equation (11). Taking the radial single-layer gradient porous structure with R = 8, *P*_in_ = 78%, *P*_out_ = 44% and $\bar{P}$ = 61% as an example, *n* = 2 is calculated according to Equation (13). The bias *C* function of the structure is obtained by substituting $P_{in}$, $P_{out}$, *n* and R into Equation (11), and the result is displayed in Equation (14). The top view of the obtained single-layer radial gradient porous structure is shown in Supplementary Figure S6.

(14) $$C(r)=0.0304r^{2}-0.9712$$

#### 2.2.4. Radial Dual-Layer Gradient Structure Design

For the same reason as for generating an axial dual-layer gradient structure, radial dual-layer gradient structure is also created. The outer radial gradient structure is developed by assigning $\phi_{P}\leq C_{3}(r)$ as solid and $\phi_{P}>C_{3}(r)$ as the porous region, and its top view is shown in Supplementary Figure S7a. The inner radial gradient structure is created by assigning $\phi_{P}\geq C_{4}(r)$ ($C_{3}(r)>C_{4}(r)$) as solid and $\phi_{P}<C_{4}(r)$ as the porous region, and its top view is displayed in Supplementary Figure S7b. Through a Boolean intersection operation on the outer and inner radial gradient structures, a radial dual-layer gradient structure (Supplementary Figure S7c) is obtained.

### 2.3. Design Cases

#### 2.3.1. Six Structure Design Cases

**Figure 2:**
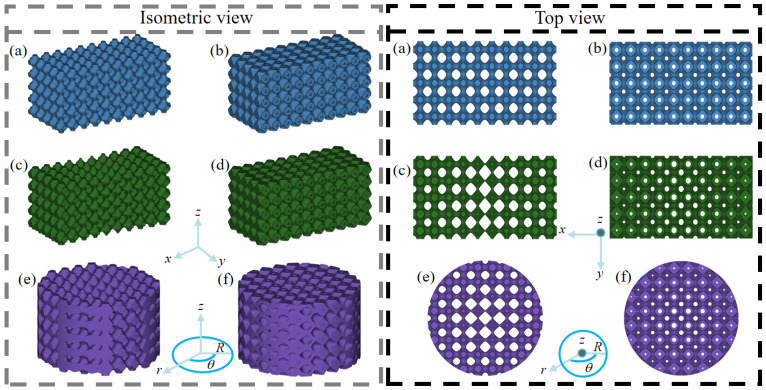
Isometric and top view of six P-type structure design cases of (**a**) HS, (**b**) HD, (**c**) ASG, (**d**) ADG, (**e**) RSG, (**f**) RDG.

In this section, six structure design cases containing both homogeneous and gradient structures are generated with the same average porosity for performance evaluation and comparison. Specifically, the six types of design are the homogeneous single-layer structure (HS), homogeneous dual-layer structure (HD), axial single-layer gradient structure (ASG), axial dual-layer gradient structure (ADG), radial single-layer gradient structure (RSG) and radial dual-layer gradient structure (RDG). The isometric and top views of the above designed structures are displayed in Figure 2a–f. Since the theoretical porosity range of the single-layer gradient structure is 22–78%, the theoretical porosity range of the dual-layer gradient structure is 44–99%. Hence, the common porosity range of 44–78% is set for all structure design cases, and each structure has the same average porosity of 61%.

The sizes of the structures are determined as follows: A TPMS cell size of 2 mm is used considering the precision of selective laser melting, which is the best manufacturing technique for such porous structures [32]. In order to avoid the cell number effect of the TPMS structure, the numbers of cells of the axial gradient structures are set to 10×5×5 (x×y×z), which are more than 4×4×4 (x×y×z), since the mechanical properties of such size tend to be stable [33]. For the same purpose of avoiding the cell number effect, radial gradient structures are designed with the same size along the z direction with a similar number of cells, which results in a radius of 8 mm and a height of 10 mm. Since natural bone has hierarchical distribution of porosity with higher porosity in the core [34], such a trend is considered in the gradient structure design. Specifically, the ADG structure is developed with the highest porosity of 39% at *x* = 0 mm and lowest porosity of 22% at *x* = 10 mm for both the inner and outer gradient structures. After Boolean intersection, the ADG structure has the highest porosity of 78% at *x* = 0 mm and the lowest porosity of 44% at *x* = 10 mm. Then, the structure is mirrored at *x* = 0 mm to create the other symmetric counterpart. Likewise, the RDG structure is generated with the highest porosity of 39% at *r* = 0 mm and the lowest porosity of 22% at *r* = 8 mm for the inner and outer gradient structures. Hence, the RDG structure possesses the highest porosity of 78% at *r* = 0 mm and the lowest porosity of 44% at *r* = 8 mm after Boolean intersection.

#### 2.3.2. Ten Structure Design Cases

**Figure 3:**
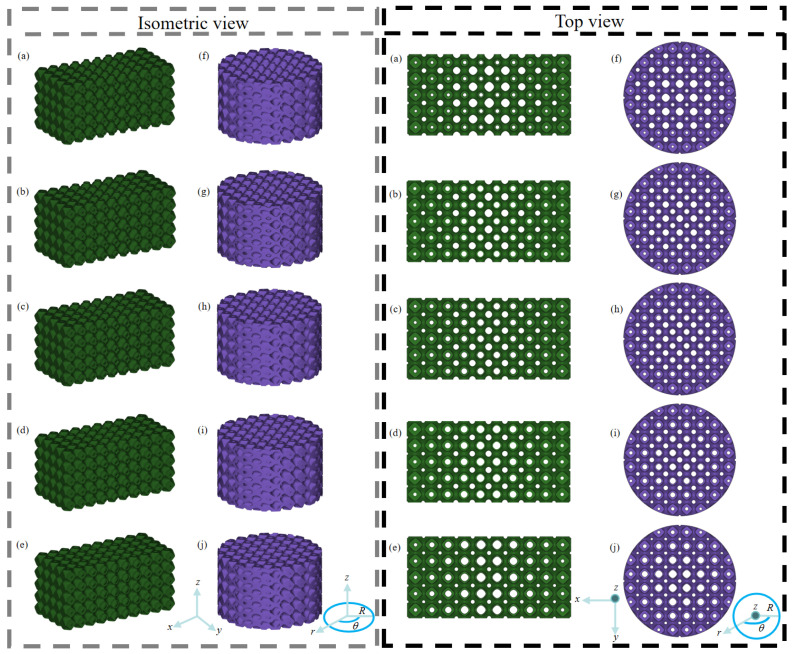
Ten dual-layer gradient structure designs (isometric view and top view): (**a**–**e**) A_1_–A_5_, (**f**–**j**) R_1_–R_5_.

In this section, various dual-layer gradient structures are designed. Recalling that the porosity range of structures should be within 50–90% for bone scaffold application, ten axial and radial dual-layer gradient structures with average porosity of 70% but different combinations of inner and outer layers are designed. Among them, half of the design cases are axial dual-layer gradient structures (A_1_–A_5_), while the other half are radial dual-layer gradient structures (R_1_–R_5_). The porosity ranges and average porosities $\bar{P}$ of the inner and outer single-layer gradient structures, as well as the dual-layer structure, are summarized in Table 1. The isometric and top views of the ten designed dual-layer gradient structures are provided in Figure 3.

### 2.4. Finite Element Model

#### 2.4.1. Mechanical Property Finite Element Model

To evaluate the mechanical behavior of the designed structures, finite element analysis (FEA) is employed. HyperMesh 2021 (Altair Engineering Inc., Troy, MI, USA) is used to generate the finite element meshes from the designed STL files, and ANSYS Workbench 2021 R2 (ANSYS Inc., Canonsburg, PA, USA) is used to perform the simulations. The finite element mesh type is Tetrahedral, and the mesh size is 0.1 mm, which result in a minimum element number of 3,013,072 for the HS structure and a maximum element number of 12,816,868 for the RDG structure. In this study, Ti-6Al-4V is selected due to its good biocompatibility with human tissues. The assumed material properties of Ti-6Al-4V are an elastic modulus of 110 GPa, a Poisson’s ratio of 0.34, a Johnson–Cook parameter (initial yield stress) of 875 MPa, and a strain hardening exponent of 0.387 [35].

The boundary conditions of the compression are set as follows: six degrees of freedom are set fixed at all nodes at the bottom of the structure, and a displacement *U_z_* of 0.5 mm (5% of height) is applied downward at all nodes on the top. The illustration of such boundary conditions is shown in Supplementary Figure S8, and the results obtained from this confined compression simulation are used to calculate the effective compressive elastic modulus (referred as elastic modulus).

#### 2.4.2. Permeability Property Finite Element Model

Permeability is the fluid’s ability to pass through porous structures [36]. In this section, fluid simulation is conducted to evaluate the permeability of ten new designed dual-layer gradient structures. To measure the permeability, fluid needs to be incompressible steady state laminar flow [37] which satisfies the Navier–Stokes equation (Equation (15)).

(15) $$\rho \left(\frac{\partial v}{\partial t}+v\cdot \nabla v\right)=\mu \nabla^{2}v-\nabla p,\;\nabla v=0$$

where ρ is density (kg/m^3^), v is velocity (m/s), μ is viscosity (Pa·s), and p is the pressure of the fluid (Pa). ∇ is the del operator. The permeability *K* (m^2^) of the structure is calculated by Darcy’s law, as shown in Equation (16) [37]:

(16) $$K=\frac{v\mu L}{\Delta p}$$

where L is the length and Δp is the pressure difference from one end to the other end of the structure that the flow encounters. Specifically, Δp was obtained based on the averaged pressure of the upper and lower end faces. The simulations were performed under laminar flows (Reynolds number < 10), and the permeability calculated using Darcy’s law is valid. COMSOL Multiphysics 6.3 (COMSOL Inc., Burlington, MA, USA) is used for fluid simulation, and some of the settings are as follows: The upper end face is set as inlet, the lower end face is set as outlet, and the rest of the surface is impermeable. The velocity of inlet flow is set to 1 mm/s, and the pressure of the outlet is set to 0 Pa [38]. Water is selected as the fluid, and a density ρ of 1000 kg/m^3^ and viscosity μ of 0.001 Pa·s are used so that laminar flow is ensured. The boundary conditions of the fluid simulation are illustrated in Supplementary Figure S9. The pressure of the inlet is solved after simulation, and the pressure difference Δp is obtained.

### 2.5. Declarations of Use of Generative AI for Figure Preparation

Generative artificial intelligence was used only for the preparation of the illustrative graphics in Figure 1b,c in the Introduction. These images were created using ChatGPT with GPT-5.4 Thinking (OpenAI) to visually depict porous morphology of femoral tissue. They were used solely for illustration and were not used for data generation, quantitative analysis, experimental measurement, or result interpretation.

## 3. Results and Discussion

### 3.1. Mechanical Property Analysis of Six Design Cases

#### 3.1.1. Stress Field Analysis of Six Design Cases

**Figure 4:**
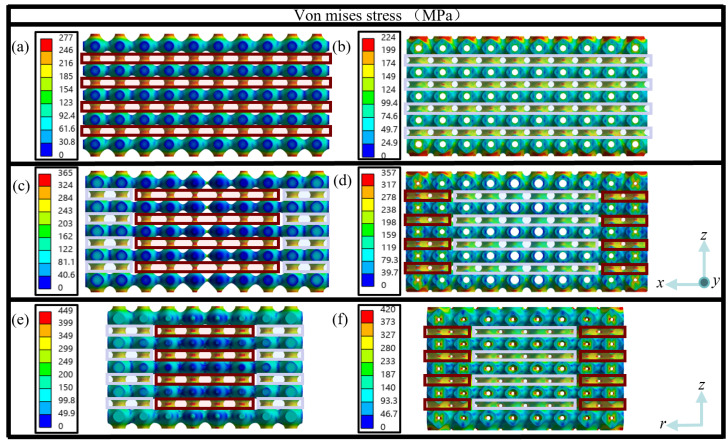
Stress fields of six structure design cases of (**a**) HS, (**b**) HD, (**c**) ASG, (**d**) ADG, (**e**) RSG, (**f**) RDG in vertical cross-sectional view at the center of the structures at strain of 0.001.

The von Mises stress (referred as stress) fields of the six structures are obtained from simulation, and the vertical cross-sectional view of the stress fields at strain of 0.001 at the center of the structure are displayed in Figure 4. It can be seen from Figure 4 that non-uniform stress distributions are found in all six design cases, the stress differences in dual-layer structures are less than single-layer structures, and the maximum stresses are mainly located at the interfaces between adjacent unit cells along loading direction.

However, the values and the regions of maximum stresses are different. The maximum stresses in both HS and HD structures appear quite uniformly at all interfaces between adjacent unit cells along loading direction, as indicated by the dark-red solid rectangles (Figure 4a) and light-indigo solid rectangles (Figure 4b), respectively. The maximum stress of HS is 277 MPa and that of HD is 224 MPa, which demonstrates that dual-layer structure distinctively reduces maximum stress for homogeneous structure. For gradient structures, the maximum stress of 365 MPa occurs in the higher-porosity regions in the center, as shown by dark-red solid rectangles, while the maximum stress in lower-porosity regions at two sides is alleviated to around 300 MPa, as highlighted within the light-indigo solid rectangles for ASG structure (Figure 4c). In contrast, the maximum stress of 357 MPa shows in the lower porosity regions at two sides, as displayed in dark-red solid rectangles, while most stresses in the higher porosity regions in the center are reduced to below 200 MPa, as highlighted by the light-indigo solid rectangles in ADG structure (Figure 4d). For RSG and RDG structures, the stress distributions are similar to those in ASG and ADG structures, but the maximum stresses are 449 MPa and 420 MPa, respectively. The above results also indicate that dual-layer structures can reduce maximum stress for gradient structures, and single-layer and dual-layer gradient structures show opposite maximum stress distributions in the high and low porous regions. In addition, the maximum stress presents the increasing trend from homogeneous to axial gradient to radial gradient structure for both single-layer and dual-layer structures.

**Figure 5:**
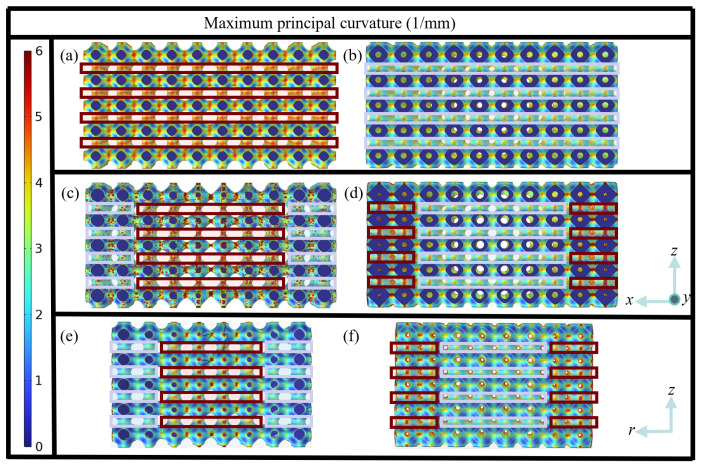
Maximum principal curvature distributions of six structure design cases of (**a**) HS, (**b**) HD, (**c**) ASG, (**d**) ADG, (**e**) RSG, (**f**) RDG, shown in vertical cross-sectional view at the center of the structure.

To better understand the maximum stress distributions within the structures, the maximum principal curvature distributions of six structure design cases are generated and shown in Figure 5. It can be seen that the maximum principal curvatures are found at cell joints, and the values of maximum principal curvature match the values of maximum stress, in which the regions with higher stresses own larger maximum principal curvature values and the regions with lower stresses possess smaller maximum principal curvature values, as indicated by the dark-red solid and light-indigo solid lines, respectively. The reason is that less maximum principal curvature means smoother geometric transitions, which will mitigate local stress concentration.

Moreover, the highest value of the maximum principal curvatures of HS, ASG and RSG structures are 5.27, 5.79 and 5.91, respectively, those of HD, ADG and RDG are 4.89, 5.51 and 5.53, respectively. This suggests that dual-layer structures have smaller maximum principal curvatures than single-layer structures, and that the maximum principal curvature exhibits an increasing trend from homogeneous to axial gradient to radial gradient structures for both single-layer and dual-layer structures, which matches the aforementioned characteristics of the maximum stress distribution.

#### 3.1.2. Yield Strength and Elastic Modulus Analysis of Six Design Cases

The yield strength and elastic modulus of six structure design cases are evaluated in this section. The nominal compressive strain *ε* is calculated as shown in Equation (17):

(17) $$\varepsilon =\frac{U}{L}$$

where *U* denotes the vertical displacement applied to the top of the structure and *L* is the height of the structure. The nominal compressive stress *σ* (MPa) is calculated by Equation (18).

(18) $$\sigma =\frac{F}{A}$$

where *F* (N) is reaction force at the bottom of the structure obtained from the simulation results, and *A* (mm^2^) is cross-sectional area of the structure. The elastic modulus *E* (GPa) of the structure is determined by Hooke’s law, as shown in Equation (19) using the stress and strain during the elastic deformation stage.

(19) $$E=\frac{\sigma }{\varepsilon }$$

**Figure 6:**
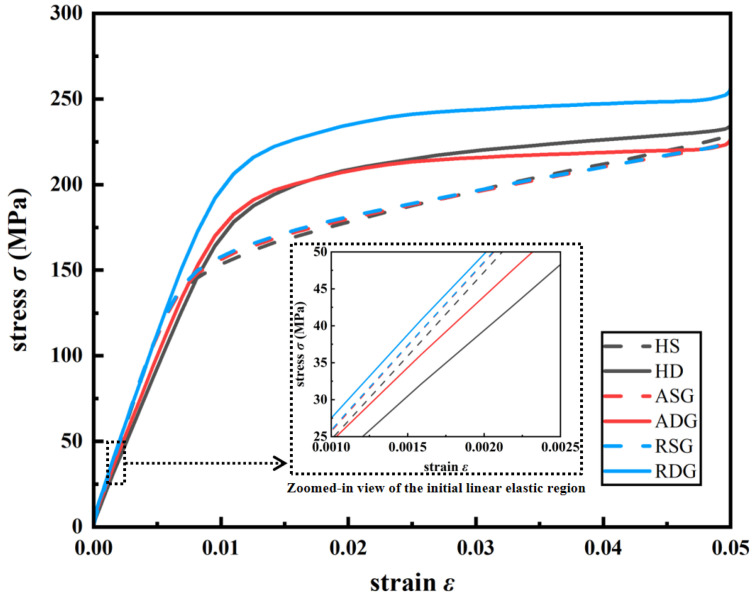
Nominal stress–strain curves of HS, HD, ASG, ADG, RSG, RDG under compression, obtained through fitting simulation results.

**Figure 7:**
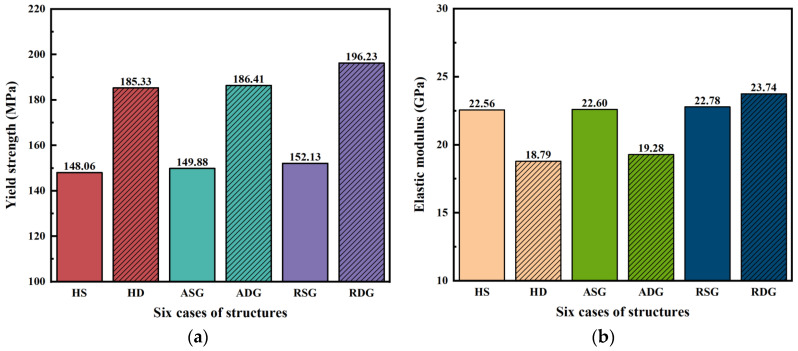
Bar plots of (a) yield strength and (b) *E* of six structure design cases, calculated from simulation results.

The nominal stress–strain curves of six design cases under compression at strains 0–0.05 from simulation are shown in Figure 6, and the yield strengths are calculated from the curves, and *E* is calculated using *F* and *σ* at an elastic strain of 0.001. The summary mechanical property results of the six design cases are presented in Table 2, and the *E* and yield strength values of six design cases are also plotted in Figure 7.

It can be seen from Figure 6 and Figure 7a and Table 2 that dual-layer structures exhibit higher yield strength than their single-layer counterparts. Specifically, the yield strength increases from 148.06 MPa to 185.33 MPa (increase of 25.2%) for homogeneous structures, from 149.88 MPa to 186.41 MPa (increase of 24.4%) for axial gradient structures, and from 152.13 MPa to 196.23 MPa (increase of 29.0%) for radial gradient structures. This enhancement is consistent with the analysis in Section 3.1.1, in which dual-layer structures smooth the geometric transition at cell interfaces and reduce the maximum principal curvature. As a result, stress concentrations are alleviated, and onset of local yielding is delayed, which eventually leads to higher yield strength. In addition, the stress–strain curves are quite similar among single-layer structures, while some differences exist for dual-layer structures (see Figure 6). Accordingly, the yield strengths only present a slight increase from HS to ASG to RSG, while the yield strengths increase a bit more from HD to ADG to RDG (see Figure 7a), which indicates that dual-layer structures are more sensitive to yield strength. Among all cases, RDG attains the highest yield strength, indicating the greatest resistance to permanent deformation.

**Figure 8:**
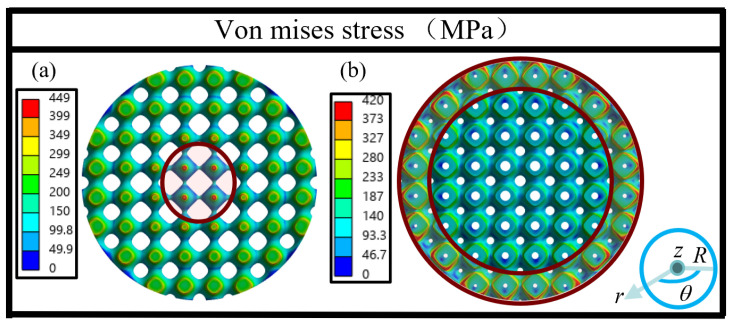
Stress fields of (**a**) RSG and (**b**) RDG (top view) at a strain of 0.001.

From Figure 6 and Figure 7b and Table 2, the values of *E* range from 18.79 to 23.74 GPa, which fall within the recommended range for bone scaffolds (10–30 GPa). For homogeneous and axial gradient designs, dual-layer structure leads to a noticeable reduction in *E*. Specifically, the *E* decreases from 22.56 to 18.79 GPa, which corresponds to a reduction of approximately 16.7% for homogeneous structures, and *E* decreases from 22.60 to 19.28 GPa, namely, about a 14.7% decrease for axial gradient structures. This may be due to the fact that the deformation mode is shifted from stretch-dominated for single-layer structures to bending-dominated for dual-layer structures [39], and according to the Gibson–Ashby model, stretch-dominated structures possess higher *E* values than bending-dominated ones for a given relative density [40]. Therefore, the *E* of HD and ADG are lower than HS and ASG. In contrast, radial gradient designs show the opposite trend since RDG has higher *E* (23.74 GPa) than RSG (22.78 GPa). The top views of the stress fields of RSG and RDG are displayed in Figure 8. It can be seen that in RDG, the outer area with lower porosity, as indicated by the area within the dark-red solid circles, bears more stress, and it acts as a hard outer shell, which may be responsible for higher *E* in RDG. Such structure is consistent with the characteristics of natural bone [41], and it is also found by another study that such structure results in higher *E* [42].

In addition, it can also be seen from Figure 7b that *E* only exhibits a slight increase from HS to ASG to RSG, while the increase in *E* is more obvious from HD to ADG to RDG (see Figure 7b), which shows that dual-layer structures are more sensitive to *E*. Moreover, it is noted that RDG has the highest *E* of 23.74 GPa among all six cases, which means the greatest resistance to elastic deformation.

Based on the analysis in Section 3.1.1 and Section 3.1.2, dual-layer gradient structures present the following advantages: (1) the pore distribution is similar to that of human bones, (2) the maximum stress can be reduced under compression, (3) the maximum stress appears at the lower porosity region at the borders under compression, which matches the characteristics of load bearing at borders in human bone, (4) elastic modulus values of 19.28–23.74 GPa are within the recommended range for bone scaffolds, (5) the higher yield strength and greater variations in both yield strength and *E* exhibit high potential for improvement. As a result, dual-layer gradient structures are preferred for bone scaffold applications, and will be chosen for further analysis in the following section.

### 3.2. Mechanical Property Analysis of Ten Design Cases

#### 3.2.1. Stress Field Analysis of Ten Design Cases

**Figure 9:**
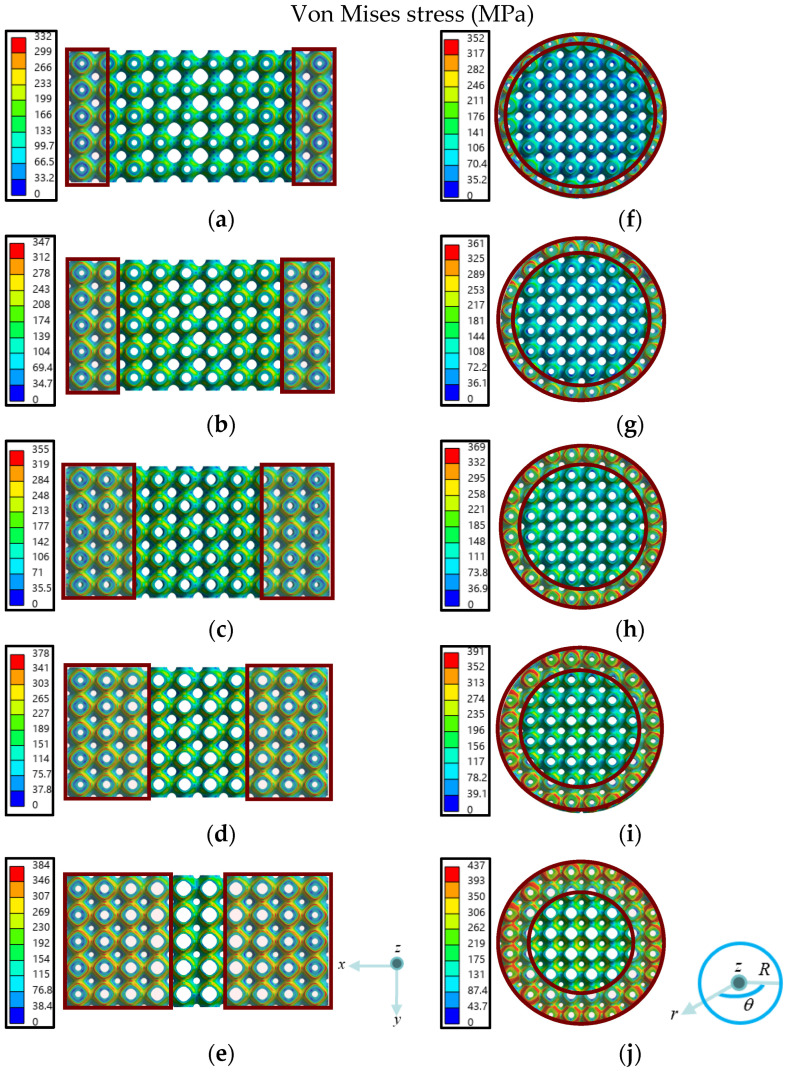
Stress fields of ten dual-layer gradient structure designs (top view): (**a**–**e**) A_1_–A_5_, (**f**–**j**) R_1_–R_5_ at strain of 0.001.

After compression simulation with same settings, the stress field results of new dual-layer gradient structures are obtained. Figure 9 shows the top view of the stress fields of the structures at strain 0.001. It can be seen that higher stresses are mainly located in the lower porosity zones, as indicated by areas within the dark-red solid lines, which matches the result of the stress field in dual-layer gradient structures in Section 3.1.1. In addition, there exist distinctive features from the newly designed structures with controlled redistribution of porosity based on different inner and outer gradient structures. First, the maximum stresses of both types of dual-layer structures increase, among which the maximum stress of axial dual-layer gradient structures increases 15.7% from 332 MPa in A_1_ to 384 MPa in A_5_, and that of radial dual-layer gradient structures increases 24.1% from 352 MPa in R_1_ to 437 MPa in R_5_. Second, higher stresses progressively expand to lower porosity regions from A_1_–A_5_ and R_1_–R_5_.

It is also noted that the maximum stresses of gradient structures with the same inner and outer layers are different: the maximum stresses of radial dual-layer gradient structures are higher than that of axial dual-layer gradient structures. Specifically, for A_1_&R_1_ and A_5_&R_5_, the differences of maximum stress are higher, which are likely due to the larger difference in $\bar{P}$ in the inner and outer layers. Comparing those two pairs, the increase of maximum stress between A_5_ and R_5_ is much greater, reaching an increase of 13.8%.

In summary, with the increase in $\bar{P}$ in the inner layer and decrease in $\bar{P}$ in the outer layer, the maximum stress increases and the regions with higher stresses expand towards inside where more porosities exist. For structure pairs with the same inner and outer $\bar{P}$ values, the maximum stresses in radial dual-layer structures are higher than those of axial dual-layer structures. Hence, it can be inferred that structures with gradient porosity along a single direction perpendicular to the load direction will alleviate maximum stress compared with structures with gradient porosities along various directions perpendicular to the load. In addition, the maximum stress difference in A_5_&R_5_ is the most distinctive.

**Figure 10:**
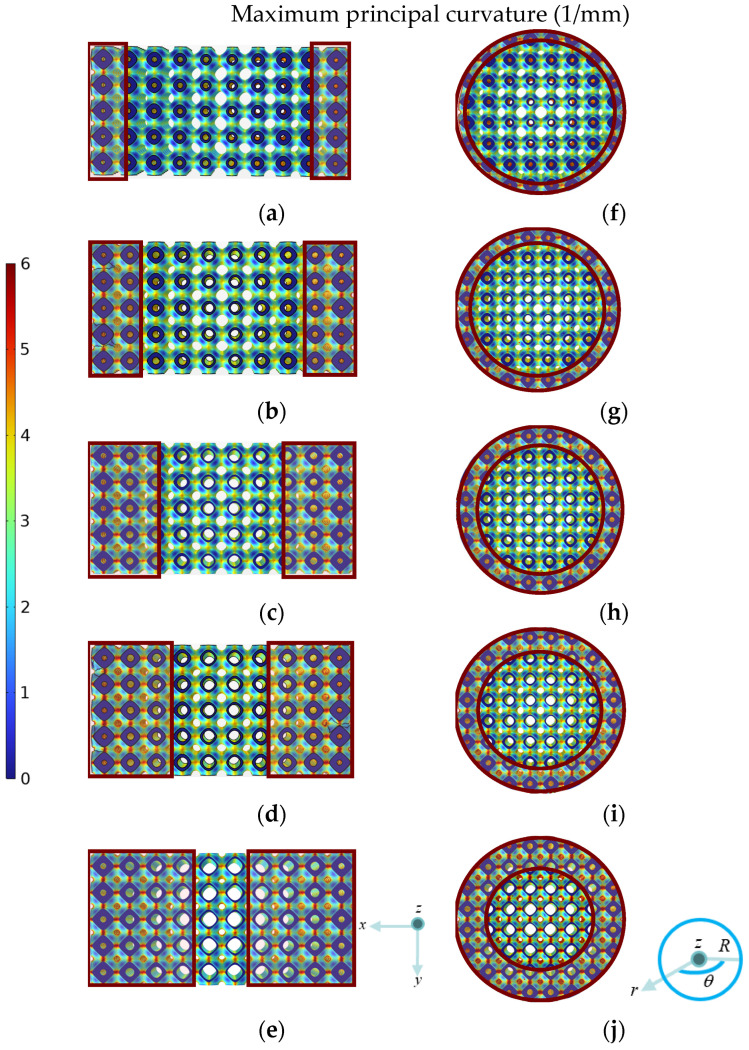
Maximum principal curvature distributions of ten dual-layer gradient structure designs (top view): (**a**–**e**) A_1_–A_5_, (**f**–**j**) R_1_–R_5_.

To further elucidate the influence of inner and outer layer porosity allocation on stress distribution, the distributions of maximum principal curvature are displayed in Figure 10. A clear correspondence between porosity and maximum principal curvature can be observed: the regions enclosed by the dark-red solid lines, which exhibit relatively large maximum principal curvature, become more pronounced as the $\bar{P}$ of inner layer gradient structure increases and the $\bar{P}$ of the outer layer gradient structure decreases. Hence, comparing Figure 9 and Figure 10, it can be found that by increasing the porosity of the inner layer gradient structure while decreasing the porosity of the outer layer gradient structure, increasingly higher maximum principal curvature occurs, eventually resulting in increasingly higher stresses, which coincides with the stress distribution in Figure 9.

#### 3.2.2. Yield Strength and Elastic Modulus Analysis of Ten Design Cases

**Figure 11:**
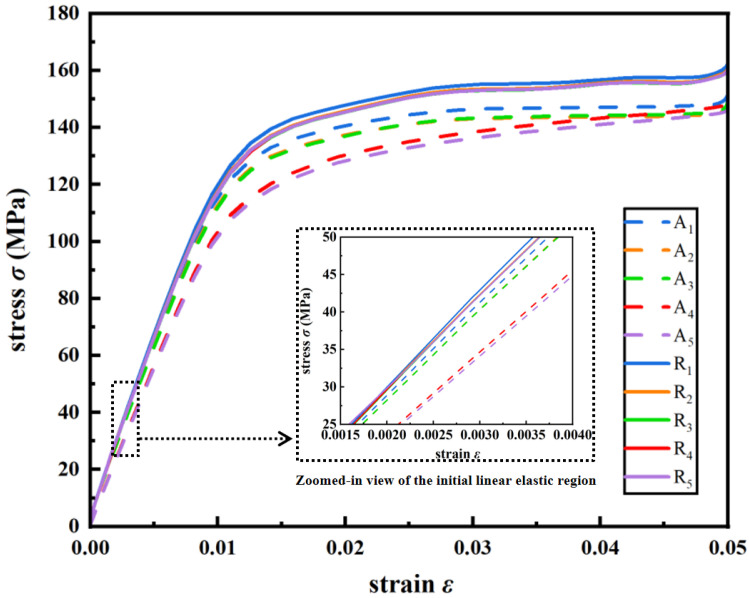
Nominal stress–strain curves of A_1_–A_5_ and R_1_–R_5_ under compression, obtained through fitting simulation results.

**Figure 12:**
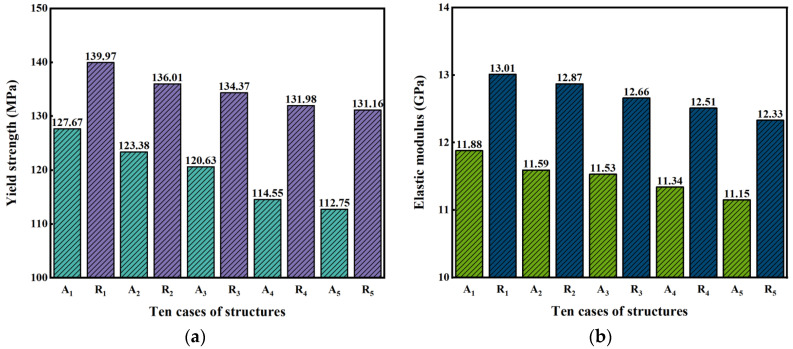
Bar plots of (**a**) yield strength and (**b**) *E* of ten dual-layer gradient structures, calculated from simulation results.

The nominal stress–strain curves of ten dual-layer gradient structures under the compression condition as shown in Figure 3 are obtained from simulation and shown in Figure 11, and the calculated *E* and yield strength are summarized in Table 3 and plotted in Figure 12. It can be seen from Figure 11 and Figure 12a and Table 3 that radial dual-layer structures (R_1_–R_5_) have higher yield strength than axial dual-layer structures (A_1_–A_5_). In addition, with increase in inner $\bar{P}$ and decrease in outer $\bar{P}$, the yield strength decreases for both types of structures. This trend is consistent with the results from Section 3.2.1, in which maximum stress and expanded area of higher stress increase with the increased inner layer porosity but reduced outer layer porosity, and such a phenomenon promotes stress concentration and earlier local yielding, and thus lowers the yield strength. However, from A_1_–A_5_, the yield strength is decreased more (127.67 MPa to 112.75 MPa), while from R_1_–R_5_, the yield strength is deceased less (139.97 MPa to 131.16 MPa). Such results imply that the changes in porosity in the inner and outer layers in axial dual-layer gradient structures are more sensitive to yield strength.

It can also be seen from Figure 12b and Table 3 that *E* values of the new dual-layer gradient structures fall in a range of 11.15 to 13.01 GPa, which are within the recommended *E* range for bone scaffolds (10–30 GPa). Moreover, it can be seen that R_1_–R_5_ exhibit higher *E* than A_1_–A_5_ with the same porosity combination, and thus it can be inferred that structures with gradient porosities along various directions perpendicular to the load direction will possess greater *E* than structures with gradient porosity along a single direction perpendicular to the load. In addition, with increasing porosity of the inner layer and decreasing porosity of the outer layer, *E* gradually decreases from 11.88 to 11.15 GPa and from 13.01 to 12.33 GPa for axial and radial structures, respectively. Hence, the porosity in the outer layer has a positive effect, while the porosity in inner layer has a negative impact, on the *E* of the overall structure. With the increasing porosity of the inner layer and decreasing porosity of the outer layer, the area with larger maximum principal curvature expands as shown in Figure 10, which amplifies local stresses and promotes stress concentration [43]. The expansion of stress-concentrated regions often reflects enhanced nodal rotation, bending moments, and shear effects, which are typical characteristics of a bending-dominated deformation mode [44]. Therefore, increasing porosity of the inner layer and decreasing porosity of the outer layer presents a more bending-dominated deformation mechanism, resulting in lower *E* according to the Gibson–Ashby model.

In summary, R_1_–R_5_ show higher yield strength and *E* than A_1_–A_5_ with the same inner and outer porosity combination, and thus structures with gradient porosities along various directions perpendicular to the load direction possess increased yield strength and *E* than structures with gradient porosity along a single direction perpendicular to the load. With increasing porosity of the inner layer and decreasing porosity of the outer layer, yield strength and *E* gradually decrease for both types of dual-layer gradient structures, which may be due to increased values and area of maximum stress and increased level of bending-dominated deformation mechanism.

### 3.3. Permeability Property Analysis of Ten Design Cases

**Figure 13:**
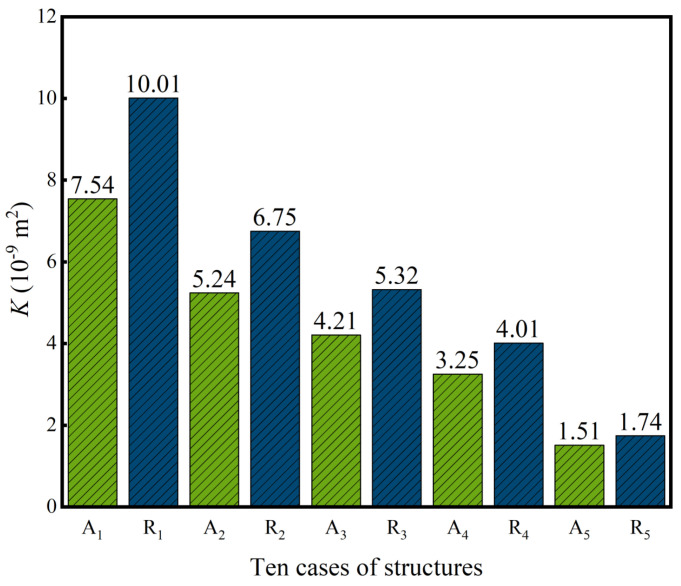
*K* of ten dual-layer gradient structures, calculated from simulation results.

The permeability *K* can be calculated according to Equation (16), and *K* values of ten dual-layer gradient structures are presented in Figure 13. It can be seen from Figure 13 that *K* values of ten structures are in the range of 1.51–10.01 × 10^−9^ m^2^, which fall within the *K* value range of bone scaffolds of 2.56 × 10^−11^–74.3 × 10^−9^ m^2^ [27]. In addition, it can be seen that the *K* values of radial dual-layer gradient structures are higher than those of axial dual-layer gradient structures. Moreover, as $\bar{P}$ of the inner layer increases and $\bar{P}$ of the outer layer decreases, *K* values as well as the differences of *K* values of two types of dual-layer gradient structures decrease. Similarly, it can be inferred that the porosity in the outer layer has a positive influence while the porosity in the inner layer has a negative effect on the *K* of the overall structure.

**Figure 14:**
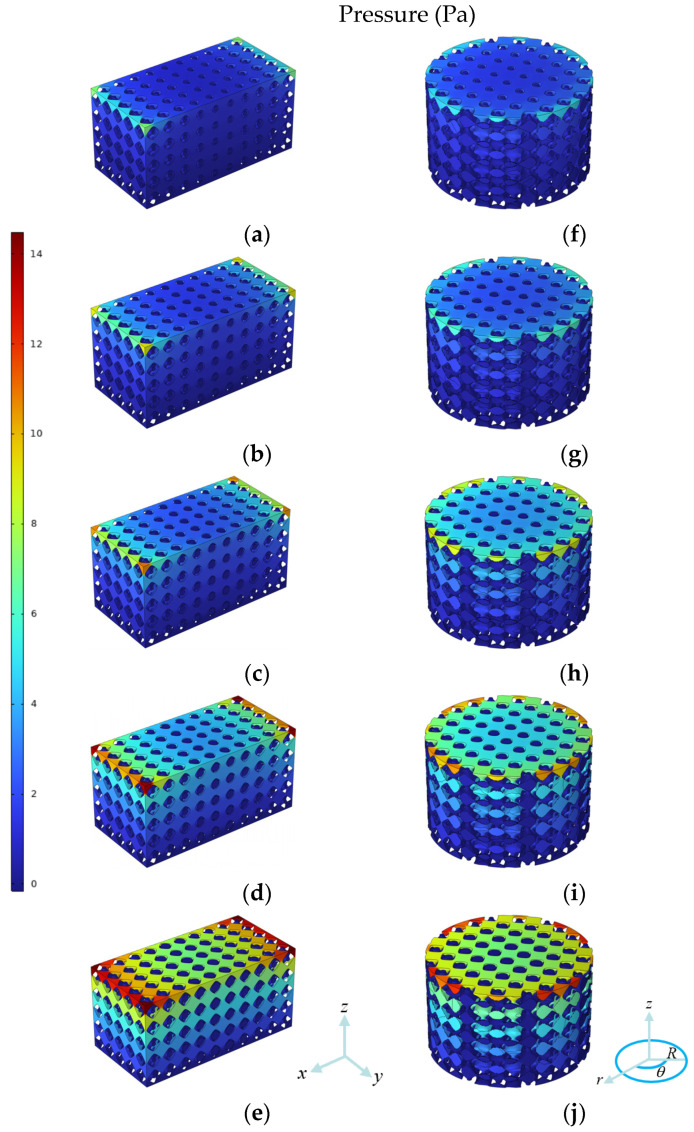
Pressure field distribution of (**a**–**e**) A_1_–A_5_ and (**f**–**j**) R_1_–R_5_.

The pressure field distributions of ten dual-layer gradient structures are shown in Figure 14. For all cases, the pressure is highest at the inlet face and lowest at the outlet face, presenting pressure gradient along the flow direction. It can be seen that the pressure gradually decreases from top to bottom, and the pressures are higher at edges with lower porosities for both types of gradient structures. From structures A_1_ to A_5_ and R_1_ to R_5_, the maximum pressure gradually increases, and the thus the porosity differences in the inner and outer layers have a non-negligible impact on pressure distribution. The structures with higher porosities in the inner layers but lower porosities in the outer layers result in much higher maximum pressure. In addition, comparing structures with same average inner and outer porosities, the maximum pressures of radial dual-layer gradient structures are generally slightly lower than those of axial dual-layer gradient structures.

For structures A_1_ and R_1_ with higher *K*, the maximum pressure near the inlet is relatively low, the pressure drop across the height of scaffold is moderate, and the pressure change across the surface at same height is relative mild. In contrast, in structures A_5_ and R_5_ with lower permeability, higher maximum pressure is developed at the inlet and penetrates towards the outlet, presenting higher pressure gradient. Since the permeability is calculated from Darcy’s law, larger pressure drop corresponds to lower permeability. Therefore, the observed decrease in *K* from A_1_ to A_5_ and from R_1_ to R_5_ is consistent with the progressively higher pressure gradients along the flow. Moreover, for the same inner and outer porosity combination, the radial designs R_1_-R_5_ exhibit lower maximum pressures and thus lower pressure drop than the axial designs A_1_–A_5_, which explains the higher permeability of radial dual-layer gradient structures than axial dual-layer gradient structures.

In addition to permeability, wall shear stress (*WSS*) caused by fluid flow is another important measure since it raises stimulatory signal for the cells [45], which leads to cell differentiation and proliferation [20]. The schematic illustration of *WSS* within walls is shown in Supplementary Figure S10; it can be seen that the tangential velocity of fluid increases with distance along normal direction *n* within the wall, forming a pronounced velocity gradient ($\frac{\partial v}{\partial n}$) and thereby generating *WSS* acting tangentially in the flow direction on the wall surface.

**Figure 15:**
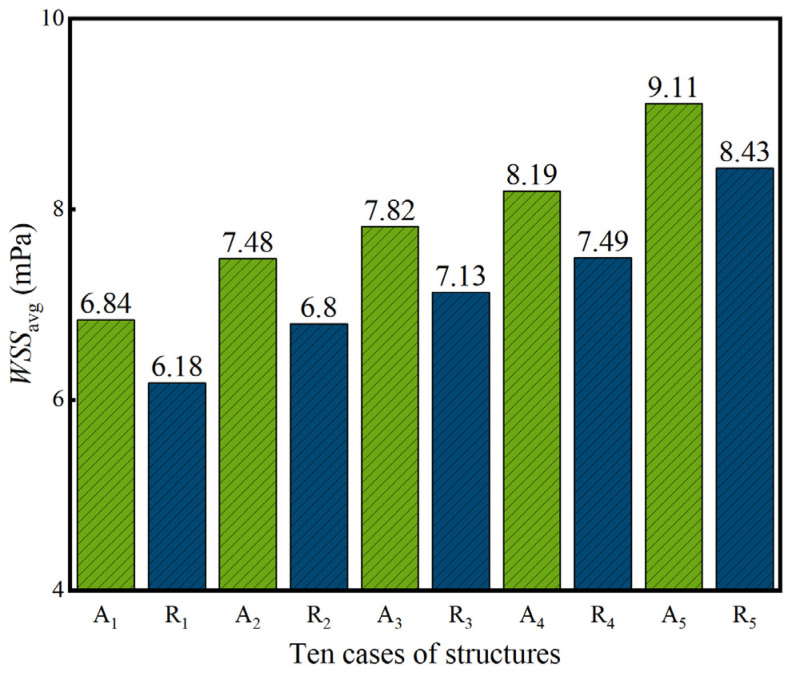
*WSS*_avg_ of ten dual-layer gradient structures, calculated from simulation results.

Under laminar flow condition, *WSS* can be defined based on the normal velocity gradient on the wall [46], and is calculated as shown in Equation (20).

(20) $$WSS=\mu \frac{\partial v}{\partial n}$$

where *n* is the normal direction at the fluid–solid interface, and the velocity gradient $\frac{\partial v}{\partial n}$ is obtained from the fluid simulation result. For *WSS*, there is no recommended optimal value [47], but it is generally accepted that *WSS* of less than 15 mPa is favorable for cell growth [48]. The average *WSSs* (*WSS*_avg_) of ten dual-layer gradient structures are obtained and shown in Figure 15. It can be seen that *WSS*_avg_ ranges from 6.18 to 9.11 mPa, which is less than 15 mPa. In addition, *WSS*_avg_ increases monotonically from A_1_ to A_5_ and from R_1_ to R_5_, which exhibit opposite trends compared to *K*. In addition, the *WSS*_avg_ values of radial dual-layer gradient structures are lower than those of axial dual-layer gradient structures with same average inner and outer porosities.

From the above results, it can be seen that radial dual-layer gradient designs possess higher *K* and lower *WSS*_avg_. It can be inferred that structures with gradient porosities along various directions perpendicular to the fluid flow present superior permeability and decreased *WSS* than structures with gradient porosity along a single direction perpendicular to the fluid flow. Among all design cases, structure R_1_ possesses highest *K* and lowest *WSS*_avg_, which demonstrates that dual-layer design with lowest $\bar{P}$ in inner layer and highest $\bar{P}$ in outer layer has superior permeability properties.

### 3.4. Comparisons with Other TPMS Gradient Structures

**Figure 16:**
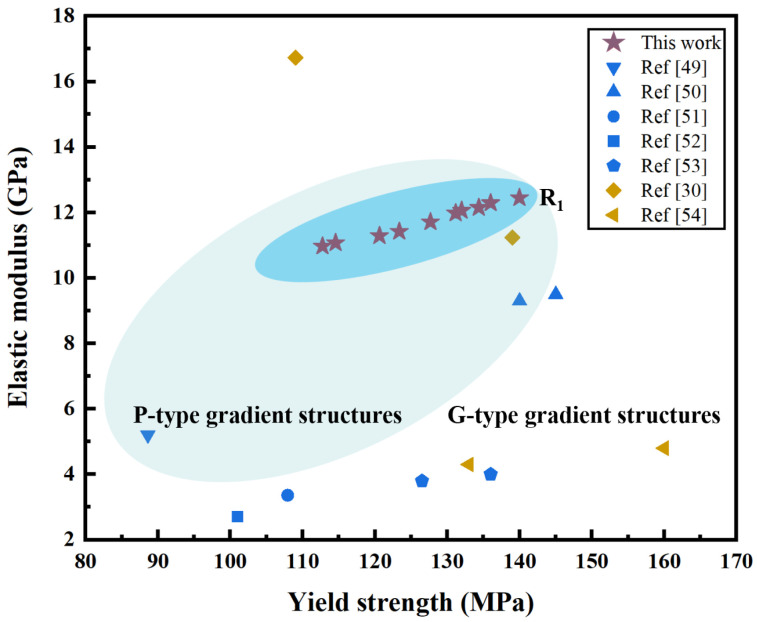
Comparison of yield strength and *E* between ten P-type dual-layer gradient structures from this study and typical TPMS gradient structures from literature.

To assess the mechanical properties of the proposed ten dual-layer gradient structures, comparisons are made with those of typical TPMS gradient structures (P-type and G-type) with the same material and similar average porosities (60% & 70%), from recent studies [30,49,50,51,52,53,54]. The comparison results of *E* and yield strength are summarized in Figure 16. The points colored in blue and mustard yellow represent structures with average porosities of 70% and 60%, respectively. It can be seen that the *E* values of dual-layer gradient structures of this work are generally higher than those of other gradient structures from the literature, and the yield strength values are moderate among all structures. Overall, the developed dual-layer gradient structures have outstanding mechanical properties with relatively high *E* and yield strength at the same time. In particular, structure R_1_ presents superior mechanical properties among all P-type gradient structures. In addition, the *E* values of the majority of the structures from literature are lower than the required range for bone scaffold application except two points from Ref. [30]. It is noted that most of the mechanical properties of dual-layer structures in this study fall within the results from Ref. [30], namely, the values of yield strength are higher while the values of *E* are lower than one of the structures in Ref. [30], and the values of yield strength are lower while the values of *E* are higher than the other structure in Ref. [30]. Such results show superior mechanical properties of the structures developed in this work since the average porosity of the structures in this study is 70%, while that of Ref. [30] is only 60%.

**Figure 17:**
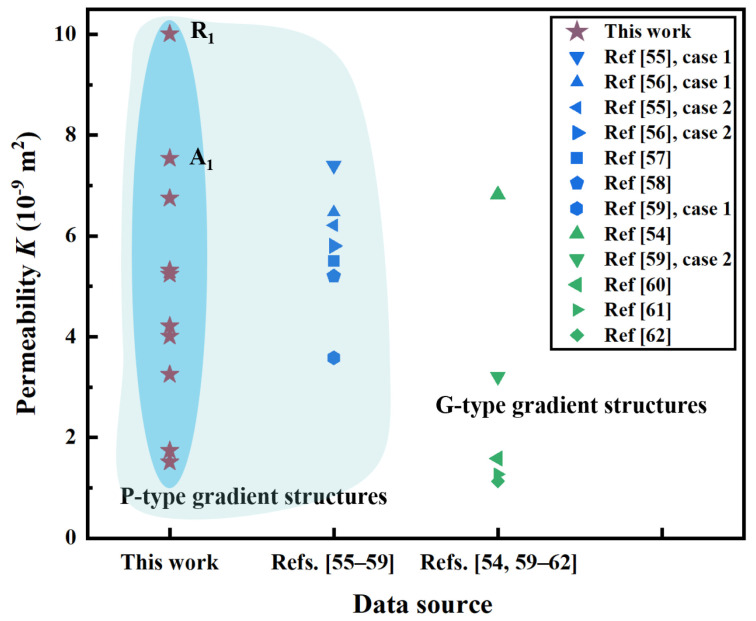
Comparison of *K* between ten P-type dual-layer gradient structures from this study and typical TPMS gradient structures from literature.

Likewise, the permeabilities of the proposed ten dual-layer gradient structures are benchmarked with that of typical TPMS gradient structures (P-type and G-type) with same average porosity from the literature [54,55,56,57,58,59,60,61,62]. The reason for selecting structures with same average porosity of 70% is that generally the permeability is higher when the porosity is higher, and it is meaningful to compare the permeabilities of our structures with structures of higher average porosity. Nevertheless, such structures with average porosity above 70% are rarely found. The comparison results of permeability *K* among structures are summarized in Figure 17. The points colored in blue and green represent P-type and G-type gradient structures, respectively. It can be seen that *K* values of P-type gradient structures are generally higher those that of G-type gradient structures. Moreover, comparing with P-type gradient structures reported in Refs. [55,56,57,58,59], the proposed P-type dual-layer gradient structures in this study exhibit comparable levels of *K*. It is worth noting that structure R_1_ achieves highest *K* and both structures R_1_ and A_1_ outperform the benchmarked P-type and G-type gradient structures, demonstrating outstanding permeability properties. Overall, the proposed dual-layer gradient structures in this study exhibit favorable mechanical and permeability performance, presenting high potential to be used in the field of bone scaffolds.

### 3.5. Limitations and Future Work

This study creatively designs the dual-layer gradient structures and evaluates the mechanical properties and permeability of the designed structures. Ti-6Al-4V is selected as the structure material due to its potential application as bone scaffolds. The limitations and future work directions are presented as follows:

Since Ti-6Al-4V is a permanent material, the pores within bone scaffolds made of Ti-6Al-4V may be gradually filled by the growth of natural bone over time. As a result, the mechanical and transport behaviors of bone scaffolds may deviate from those of the initially designed state. The long-term evolution associated with bone ingrowth was not modeled in the present work. Future studies may incorporate bone ingrowth to evaluate stage dependent mechanical and permeability behaviors.

Pore size is also an important geometric parameter affecting permeability and biological responses, and a commonly recommended pore size range of approximately 600 to 900 μm for bone scaffolds has been reported [9]. Pore size was not treated as an independent design factor while different gradient structures with rational porosity range and same overall average porosity were developed in this study. The size and distribution of pores can be considered as independent design factors for further study and comparison in the future.

The proposed axial and radial dual-layer gradient structures employ an in-plane gradient porosity distribution at the cross-section, with higher porosity in the center and lower porosity in the periphery. This design is characterized by peripheral load-bearing stability and internal enhanced permeability. However, the directionality of vascularization and tissue ingrowth were not discussed. The bone healing process and the coupling between structural gradient orientation and vascularization/bone ingrowth pathways can be studied in the future.

## 4. Conclusions

The structures of bone scaffolds need to be well designed in order to meet the application requirements. P-type TPMS is chosen for structure design in this study, and innovative dual-layer structures are proposed. The six cases of homogeneous single/dual-layer structures, axial single/dual-layer gradient structures, and radial single/dual-layer gradient structures with the same average porosity are developed first, the stress field and mechanical properties are evaluated, and the dual-layer gradient structures are determined as preferred candidates. Afterwards, ten new axial and radial dual-layer gradient structures with the same average porosity but different combinations of inner and outer structures are further constructed, and both mechanical and permeability properties are investigated. The main conclusions are the following:

(1) Among six homogeneous single/dual-layer, axial and radial single/dual-layer gradient structures, dual-layer structures possess reduced maximum stress, higher yield strength and greater variations in yield strength and elastic modulus *E*. Dual-layer gradient structures also present more similar pore and stress distributions compared to human bones, and therefore dual-layer gradient structures are chosen for further design and analysis.
(2) The mechanical and permeability properties of ten axial and radial dual-layer gradient structures meet the requirements of bone scaffold: yield strength reaches 112.75–139.97 MPa, *E* ranges from 11.15 to 13.01 GPa, permeability *K* is within 1.51–10.01 × 10^−9^ m^2^, and the average wall shear stress *WSS*_avg_ varies between 6.18 and 9.11 mPa. The mechanical and permeability properties of the dual-layer gradient structures in this study are superior when compared with other TPMS gradient structures from the literature.
(3) For ten axial and radial dual-layer gradient structures, the yield strength and *E* of radial dual-layer gradient structures are higher than those of axial dual-layer gradient structures, and axial dual-layer gradient structures are more sensitive to those mechanical properties. Moreover, with increase in inner $\bar{P}$ and decrease in outer $\bar{P}$, the yield strength and *E* gradually decrease for both types of structures due to the increased maximum stress accompanied by the expanded area of higher stress and a more bending-dominated deformation mode, respectively.
(4) Comparing five radial dual-layer gradient structures to the axial dual-layer gradient structure counterparts, permeability *K* is higher and average wall shear stress *WSS*_avg_ is lower. Moreover, *K* gradually decreases and *WSS*_avg_ progressively increases as $\bar{P}$ of the inner layer increases and $\bar{P}$ of the outer layer decreases. Increasing porosity of the inner layer and decreasing porosity of the outer layer raises more pressure gradients along the flow, which leads to decrease in *K*.
(5) Among all dual-layer gradient designs, the radial dual-layer structure with the lowest porosity of 27.5% in the inner layer and the highest porosity of 42.5% in the outer layer exhibits the highest yield strength of 139.97 MPa, *E* of 13.01 GPa and *K* of 10.01 × 10^−9^ m^2^ but the lowest *WSS*_avg_ of 6.18 mPa. Radial dual-layer gradient structure presents superior mechanical and permeability properties to axial dual-layer gradient structure, and thus has high potential in the bone scaffold application.
(6) The change of inner and outer structures and the corresponding monotonic change results of yield strength, *E*, *K* and *WSS*_avg_ provide design flexibility and insights to match structure and performance for tailored applications.

## Figures and Tables

**Table 1:** New design cases of dual-layer gradient structures.

Design Case	Inner Structure		Outer Structure		Dual-Layer Structure	
	*P* Range (%)	$\bar{P}$ (%)	*P* Range (%)	$\bar{P}$ (%)	*P* Range (%)	$\bar{P}$ (%)
A_1_ & R_1_	25–30	27.5	25–60	42.5	50–90	70
A_2_ & R_2_	25–40	32.5	25–50	37.5	50–90	70
A_3_ & R_3_	25–45	35.0	25–45	35.0	50–90	70
A_4_ & R_4_	25–50	37.5	25–40	32.5	50–90	70
A_5_ & R_5_	25–60	42.5	25–30	27.5	50–90	70

**Table 2:** Summary mechanical property results of six design cases, calculated from simulation results.

Design Case	HS	HD	ASG	ADG	RSG	RDG
*E* (GPa)	22.56	18.79	22.60	19.28	22.78	23.74
Yield strength (MPa)	148.06	185.33	149.88	186.41	152.13	196.23

**Table 3:** Summary mechanical property results of ten dual-layer gradient structure design cases, calculated from simulation results.

Design Case	A_1_	A_2_	A_3_	A_4_	A_5_	R_1_	R_2_	R_3_	R_4_	R_5_
*E* (GPa)	11.88	11.59	11.53	11.34	11.15	13.01	12.87	12.66	12.51	12.33
Yield strength (MPa)	127.67	123.38	120.63	114.55	112.75	139.97	136.01	134.37	131.98	131.16

## Data Availability

The original contributions presented in the study are included in the article, and further inquiries can be directed to the corresponding author.

## Supplementary Materials

The following supporting information can be downloaded at: https://www.mdpi.com/article/10.3390/jfb17030144/s1, Figure S1: A typical P-type cell structure. Figure S2: P-type structures with defects. Figure S3: The relationship of bias *C* and porosity *P*. Figure S4: Axial single-layer gradient P-type structure. Figure S5: Development of P-type axial dual-layer gradient structure. Figure S6: Radial single-layer gradient structure. Figure S7: Development of P-type radial dual-layer gradient structure. Figure S8: Boundary conditions of the structures under confined compression in finite element models. Figure S9: Boundary conditions of the structures in fluid simulation. Figure S10: Schematic illustration of WSS.
