# Supplementary material for: Design and Performance Evaluation of TPMS-Based Dual-Layer Gradient Porous Structures for Bone Scaffolds
Source: J Funct Biomater. 2026 Mar 13;17(3):144. doi: 10.3390/jfb17030144 (PMC13028166; doi:10.3390/jfb17030144)
Supplement: Supplementary file 1 [file jfb-17-00144-s001.zip › jfb-4143947-supplementary.pdf]

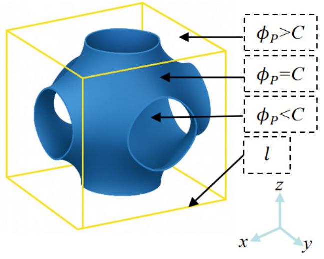

Figure S1. A typical P-type cell structure.

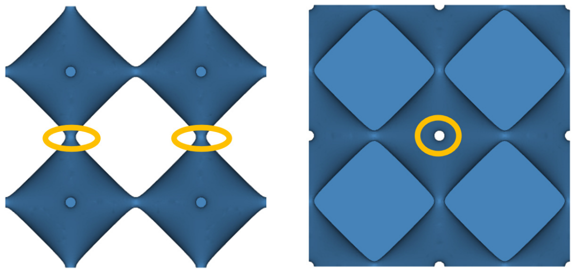

(**a**) (**b**)

Figure S2. P-type structures with defects of (**a**) pinch-off and (**b**) closed pore channel.

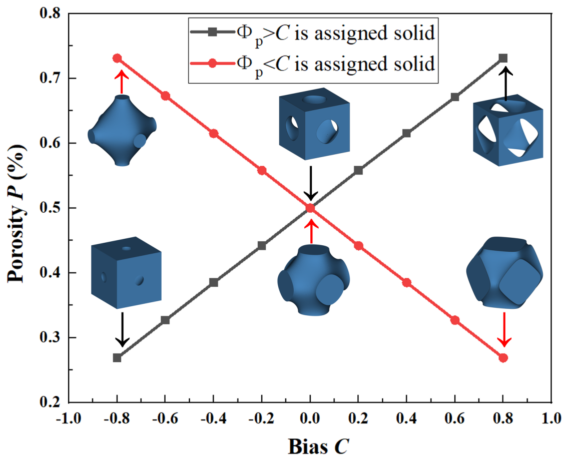

Figure S3. The relationship of bias C and porosity P.

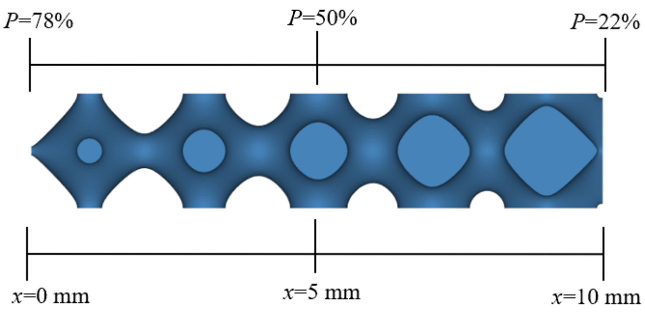

Figure S4. Axial single-layer gradient P-type structure.

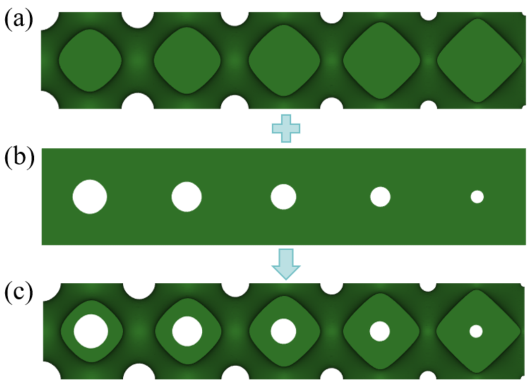

Figure S5. Development of P-type axial dual-layer gradient structure (**a**) outer axial gradient structure, (**b**) inner axial gradient structure, (**c**) axial dual-layer gradient structure.

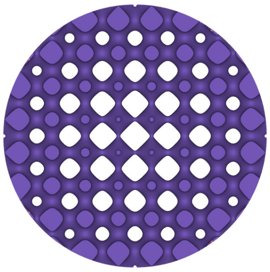

Figure S6. Radial single-layer gradient structure with P_{in} = 78%, P_{out} = 44%, n = 2 and R = 8 (top view).

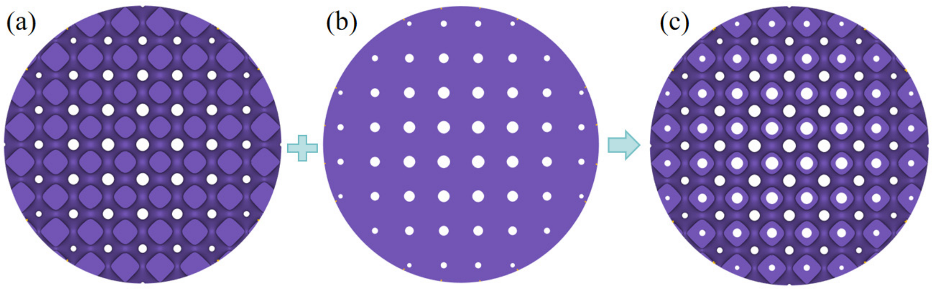

Figure S7. Development of P-type radial dual-layer gradient structure: (**a**) outer radial gradient structure, (**b**) inner radial gradient structure, (**c**) radial dual-layer gradient structure (top view).

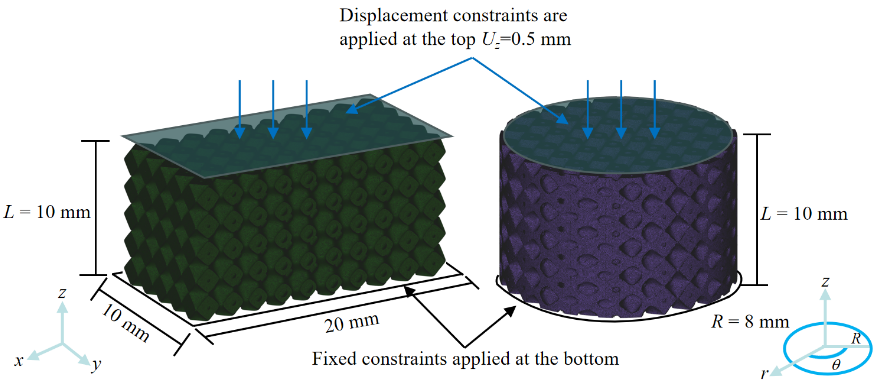

Figure S8. Boundary conditions of the structures under confined compression in finite element models.

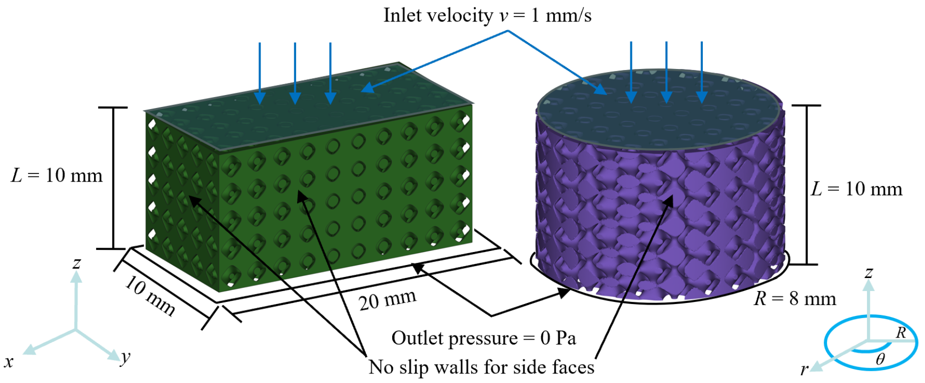

Figure S9. Boundary conditions of the structures in fluid simulation.

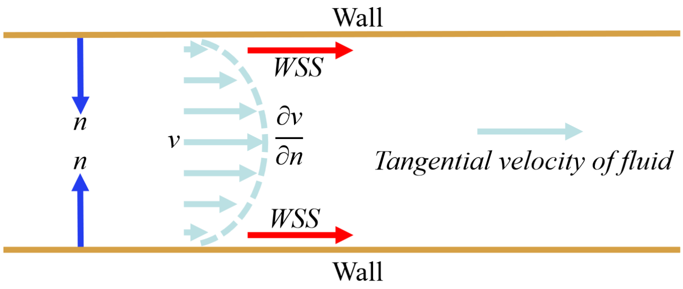

Figure S10. Schematic illustration of *WSS*.
